# Molecular Characterisation and Antibody Response to Bovine Respiratory Syncytial Virus in Vaccinated and Infected Cattle in Turkey

**DOI:** 10.3390/pathogens13040304

**Published:** 2024-04-08

**Authors:** Ozge Aydin, Aysun Yilmaz, Nuri Turan, Juergen A. Richt, Huseyin Yilmaz

**Affiliations:** 1 Department of Virology, Veterinary Faculty, Istanbul University-Cerrahpasa, Hadimkoy, 34500, Buyukcekmece, Istanbul 66506, Turkey; ozge.aydin@iuc.edu.tr (O.A.); aysunyilmaz@iuc.edu.tr (A.Y.); nturan@iuc.edu.tr (N.T.); 2 Department of Diagnostic Medicine and Pathobiology, College of Veterinary Medicine, Kansas State University, Manhattan, NY 66506, USA; jricht@vet.k-state.edu; 3 Department of Veterinary Tropical Diseases, Faculty of Veterinary Science, University of Pretoria, Onderstepoort 0110, South Africa

**Keywords:** bovine respiratory syncytial virus, antibody, ELISA, PCR, phylogenetic, cattle, Turkey

## Abstract

Bovine respiratory syncytial virus (BRSV) is one of the most important respiratory pathogens of cattle. In this study, frequency of infection, analysis of variants, and the immune status of vaccinated and non-vaccinated cattle were studied. Blood (*n* = 162) and nasal/oropharyngeal (*n* = 277) swabs were collected from 62 cattle herds in Turkey. Lung samples (*n* = 37) were also taken from dead animals and abattoirs. Antibodies to BRSV were detected in 76 (46%) out of 162 sera. The antibody levels in the vaccinated and non-vaccinated groups were statistically significant. Among 277 nasal/oropharyngeal swabs and 37 lungs, ten nasal/oropharyngeal and four lung samples were positive for BRSV-RNA. BRSV-G gene sequences of 5 out of 14 RT-PCR positive samples showed that all viruses clustered as Group-III in phylogenetic analysis with 88–100% homology. Similarity with previous Turkish BRSVs was 89–98%, and that with BRSVs detected in the USA and Czechia was 89.47–93.12%. BRSV continues to circulate in Turkish cattle, and vaccination seems beneficial in preventing BRSV. The diversity of the BRSVs found in this study needs be considered in vaccination strategies.

## 1. Introduction

Bovine respiratory syncytial virus (BRSV), also called bovine *orthopneumovirus*, is one of the causative agents of bovine respiratory disease complex (BRDC), which causes significant economical losses for the cattle industry due to severe respiratory disease leading to illness and even death as well as associated treatment and preventive biosecurity costs [1,2,3]. BRSV is an enveloped negative-sense single-stranded RNA virus and a member of the *Orthopneumovirus* genus within the family *Pneumoviridae* (International Committee on the Taxonomy of Viruses, ICTV). The non-segmented BRSV genome consists of ten genes that encode eleven proteins. The genome has nine structural proteins, namely, the attachment glycoprotein (G), fusion protein (F), RNA polymerase (L), matrix protein (M), nucleoprotein (N), polymerase cofactors M2-1 and M2-2, phosphoprotein (P), small hydrophobic protein (SH), and the two non-structural proteins NS1 and NS2 [4,5]. Phylogenetic analysis of the G and F glycoproteins indicate that there are ten subgroups for BRSV at present [6,7,8].

Cattle are the natural hosts and the reservoir for BRSV; the presence of infectious virus or antibodies to BRSV has been documented in sheep, goats and wild ruminants as well [9,10,11]. BRSV can infect cattle of all ages and breeds, although the highest incidence of severe disease is seen in young calves under six months of age. The main clinical signs observed in young calves are fever, coughing, decreased feed intake, increased breathing rate, oedema, and nasal discharge. BRSV infection predisposes cattle to secondary bacterial infections (e.g., *Mannheimia haemolytica*, *Pasteurella multocida*, *Histophilus somni* and/or *Mycoplasma bovis*) that may result in pneumonia; mortality rates in calves can be up to 20% [12,13]. Adult cattle often experience subclinical infection; such animals are the main source of virus spread. BRSV is mainly transmitted by direct contact, aerosols, and fomites, often anthropogenic [14,15]. BRSV outbreaks occur more frequently in the autumn and winter [16]. Shipping of animals, husbandry, age, and abrupt feed modifications can influence the onset and severity of BRD [17]. Vaccination and biosecurity measures to keep BRD-associated pathogens, including BRSV, out of cattle barns are effective strategies for preventing BRSV infection and the BRD complex [18,19].

Phylogenetic studies using the BRSV-F and BRSV-G gene sequences reported a total of ten genotypically different subgroups of BRSV [7,8,20]. BRSV Subgroup-I includes genotypes identified from the initial isolation of BRSV in 1980 [21,22,23]. The presence of the BRSV Subgroup-I in cattle was last reported in Belgium in 1997 [24]; since the 2000s, no Subgroup-I has been reported [25]. BRSV Subgroup-II includes genotypes detected in northern European countries such as Denmark, Sweden, and Norway; some of the BRSV genotypes detected in Japan have been reported to belong to Subgroup-II as well [20,25]. BRSV Subgroup-III, including the strains detected in Turkey, mainly includes genotypes reported from the USA [20,26,27]. BRSV Subgroup-IV is divided into two subclasses, called IA and IB, as reported by Valarcher and colleagues [20]. BRSV Subgroup-IV subclass-IA includes the BRSV Dorset and Snook genotypes isolated in England in 1971 and 1976, while Subgroup-IV subclass-IB includes BRSV genotypes isolated in the Netherlands in the 1980s [20]. BRSV Subgroup-V and Subgroup-VI have been detected in Western European countries, including Belgium and France [20,25]. In a study conducted in Croatia in 2018, two new subgroups Subgroup-VII and Subgroup-VIII, were identified by molecular diagnostic tests, including phylogenetic analyses targeting the BRSV-G gene [7]. These two subgroups were found to be similar to the BRSV genotypes detected in Italy [6]. As a result of phylogenetic analyses conducted by Kumagai and others [8] using primers targeting the BRSV-G gene, two more subgroups (IX, X), were identified. In summary, BRSV genetic diversity includes a total of ten different subgroups at present.

Vaccination against BRSV is a critical component of comprehensive herd health management strategies aimed at minimizing the impact of respiratory diseases in cattle. Vaccination protocols for BRSV are tailored to specific herd health management practices, and may vary based on factors such as the age of the cattle, their reproductive status, and the level of risk of exposure to BRSV. BRSV vaccines come in various forms, including modified live virus (MLV) vaccines and inactivated (killed) virus vaccines; each type has its own advantages and considerations. The efficacy of BRSV vaccines in preventing disease depends on several factors, including the timing of vaccination, the health status of the cattle at the time of vaccination, and the presence of maternal antibodies in calves. Vaccinated animals develop antibodies against BRSV-specific antigens, which can be detected in serological tests such as ELISA and virus neutralization [2,4,18].

Virus isolation as well as both serological and molecular techniques have been used for the detection and identification of BRSV [28,29,30]. BRSV has been isolated from many countries in Europe, America, Asia, and Africa including Turkey [31,32,33,34,35]. The majority of studies in Turkey have been aimed at determining the seroprevalence of BRSV among cattle herds, and there is limited knowledge available about sequence and phylogenetic analyses of BRSV [27,36,37]. The isolation of the virus using cell cultures is rather difficult; in addition, two samples separated by an interval of 2–3 weeks need to be taken for the antibody response to distinguish between infected and vaccinated animals. Therefore, in this study we have focused on frequency, molecular detection, and sequencing of the virus to help in identifying circulating BRSV genotypes and variants in Thrace district, Turkey. We investigated the immune status of infected and vaccinated animals by detecting specific antibodies to BRSV. Because livestock farms contain high numbers of animals, serology, especially ELISA, is a cost effective way to screen antibody response to vaccination and herd exposure to infections. Knowing the antibody status of the herd helps to reduce the risk of virus transmission within and among herds and during transport of animals. Sequencing of BRSV genes in previous studies mostly targeted the F and G genes [29,35], as these genes are involved in fusion and antibody response to BRSV. As mentioned above, rapid diagnosis of BRSV by real time RT-PCR, partial amplification of the BRSV-G gene by RT-PCR, sequence and phylogenetic analyses, and comparison with other BRSVs in GenBank from both our country and other countries are valuable inputs to aid in understanding the genotypes and variants of circulating BRSV strains in Turkey.

## 2. Materials and Methods

### 2.1. Study Design

The objectives of this study were as follows: (i) to estimate the frequency and immune status of BRSV in vaccinated and non-vaccinated cattle herds from the Thrace district in Turkey reporting outbreaks of respiratory clinical signs in calves; and (ii) to determine the genotypes of circulating BRSV strains. The area of investigation included the European part of Turkey, which shares borders with Greece and Bulgaria. Dairy cattle farming is an important part of Thrace’s economy, with many small family-owned herds. As an etiological agent of BRD in young cattle, the prevalence of BRSV infection can be very high [20]; for the purposes of this study, we investigated BRSV infection in 62 herds, assuming that the virus would be present in 80% (±10) of the herds. The herds were selected randomly from among a list of herds with clinical signs provided by veterinary clinical practitioners.

### 2.2. Sampling and Study Population

Sixty two (62) cattle herds located in Thrace district, which borders the European Union, were visited between January 2019 and March 2022 during the autumn and winter seasons. Calves and young cattle 2 to 12 months old that showed signs of respiratory disease were sampled (Table 1). Blood samples were taken into tubes without EDTA for ELISA along with nasal or oropharyngeal swabs for PCR and virus isolation (Table 1). In addition, lung tissue samples were taken from animals in abattoirs and post-mortem from necropsied animals with severe respiratory disorders, including pneumonia (Table 1). A total of 162 blood, 277 swab, and 37 lung samples were collected. Swabs and lung samples were taken into tubes containing transport medium (Gibco, Dulbecco’s minimum essential medium-DMEM, Cat. No: 41966-029, New York, NY, USA) with 5% penicillin/streptomycin (Gibco, Cat. No: 15070063, New York, NY, USA) and carried to the laboratory in cold storage/4–8 °C (Table 1). Samples were either processed directly or kept in refrigerator until the next day to complete the sample processing.

### 2.3. Extraction of RNA and Reverse Transcription

Viral RNA was extracted from swabs and tissue samples using the PureLink™ RNA Mini Kit (Invitrogen, Cat. No: 12183018A, Austin, TX, USA) as described by the manufacturer. Complementary DNA (cDNA) was generated using the HighCapacity cDNA reverse transcription kit (Applied Biosystems, Cat. No: 4368814, Vilnius, Lithuania) following the manufacturer’s protocol and kept at −80 °C until required.

### 2.4. Determination of RNA Concentration and Purity

The amount of RNA in the extracted material was measured using a NanoDrop spectrophotometer (NanoDrop 1000c, Thermo Scientific, Waltham, MA, USA). The RNA concentrations and purity ratios (A_260_/A_280_) were determined using Nanodrop. A_260_/A_280_ values of high purity RNA are expected to be 1.8–2.1, though this may change depending on the structure of sample [38].

### 2.5. PCR Inhibitors of RNA Extracts

The presence or absence of PCR inhibitors can be verified by testing different dilutions prepared from RNA extracts. For this purpose, RNA was diluted 1:10 and β-actin analysis was performed by SYBR-Green real time RT-PCR using a real-time PCR instrument (Thermo Fisher Scientific, Appliedbiosystems, StepOnePlus, Waltham, MA, USA). The primers used in this study were controlled with SnapGene 6.0.2 (GenBank Accession Number: AY141970.1). Standardized SYBR Green real time RT-PCR consisted of 12.5 μL Maxima Probe/ROX qPCR Master Mix (Thermo Scientific 2× Master Mix, Cat. No: K0231, Vilnius, Lithuania), 1 μL of each primer (400 nM), 0.5 μL SYBR Green (AAT Bioquest, Cat. No: 17591, Pleasanton, CA, USA), and 0.25 μL MgCl_2_ (25 mMol) (Thermo Scientific, Cat. No: R0971, Vilnius, Lithuania) in a final volume of 25 μL PCR reaction. Cycling conditions were at 95 °C for 10 min followed by 45 cycles of 95 °C for 15 s, 63 °C for 20 s, and 72 °C for 15 s, and the melting curve was set from 60 °C to 90 °C, increasing 1 °C each 10 s. The results were evaluated according to the following acceptance criteria [38]: i. The difference between the theoretical Ct value and Ct value of the sample should be <0.5; ii. The average value of linear regression (R^2^) shall be ≥0.981; and iii. The average value of the slope of the standard curve shall be in the range of −3.6 ≤ slope ≤ −3.1.

### 2.6. Detection of BRSV by Real Time RT-PCR

For BRSV detection, previously published primers and the TaqMan probe targeted to the nucleoprotein (N) gene of BRSV were used [39]. The sequences of the primers and probe are provided in Table 2. For real-time RT-PCR, a total volume of 25 μL PCR mixture containing 12.5 μL Maxima Probe/ROX qPCR Master Mix (Thermo Scientific 2X Master Mix, Cat. No: K0231, Vilnius, Lithuania), 2 μL of each specific primer (10 μM), 1 μL of TaqMan probe (10 μM), 1 μL cDNA, and 6.5 μL nuclease free water was used. For all PCR reactions, nuclease-free water was used as negative control in place of template. Positive controls were obtained from samples submitted to the Department of Virology, Veterinary Faculty of Istanbul and previously confirmed to be BRSV positive by PCR and sequence analyses. All amplifications were performed using a real-time PCR instrument (Thermo Fisher Scientific, Appliedbiosystems, StepOnePlus, Waltham, MA, USA). Cycling conditions were at 95 °C for 10 min followed by 45 cycles of 95 °C for 15 s and 59 °C for 60 s.

### 2.7. Sequencing and Phylogenetic Analysis

Samples found to be positive by real time RT-PCR were subjected to RT-PCR for sequencing. For this, BRSV glycoprotein G (gG) gene was partially amplified by using previously published method and primers (Table 2) [29]. A total volume of 25 μL reaction mixture composed of 12.5 μL Maxima Probe/ROX qPCR Master Mix (Thermo Scientific 2X Master Mix, Cat. No: K0231, Vilnius, Lithuania), 1.25 μL (10 μM) each primer, 2 μL MgCl_2_ (25 mMol) (Thermo Scientific, Cat. No: R0971, Vilnius, Lithuania), 2 μL bovine serum albumin (Bioshop, Cat. No: ALB001.100, Burlington, ON, Canada), 2 μL of cDNA, and 4 μL nuclease-free water was used to amplify 603 bp of BRSV partial G gene. For all PCR reactions, nuclease-free water was used as negative control in place of template. Positive controls were obtained from samples submitted to the Department of Virology, Veterinary Faculty of Istanbul and previously confirmed to be BRSV positive by PCR and subsequent sequencing. All amplifications were performed in a PCR instrument (Thermo Fisher Scientific, Appliedbiosystems, StepOnePlus, Waltham, MA, USA). Cycling conditions were at 95 °C for 10 min followed by 35 cycles of 94 °C for 45 s, 50 °C for 45 s, 72 °C for 1 min, and finally one cycle at 72 °C for 7 min.

Amplified PCR products were sent to a commercial company for sequencing, and the PCR products were purified before sequencing (MedSantek, Istanbul, Turkey). The sequencing data of partial G genes (603 bp) of BRSV were edited using Chromas Pro and aligned using MAFFT version 7 (online version) [40], using multiple alignments of partial G gene sequences of BRSV data available from the National Centre for Biotechnology Information to compare the genotypic relationship between the BRSV strains in this study and other BRSV strains detected in different countries. The Maximum Likelihood:RAxML method was used to construct the phylogenetic tree with 1000 Bootstrap replicates using MegAlign Pro Software (DNASTAR, Version 17, Goettingen, Germany). The same software was used to determine the percentage homology between the BRSV strains. Five field strains of BRSV detected in this study were submitted to GenBank under the submission numbers OQ736254, 743526, OQ789520, OQ689745, and OQ713830.

### 2.8. ELISA

The sera collected from cattle were tested for the presence of antibodies to BRSV using commercial indirect ELISA kits (Bio-X Diagnostics, Monoscreen Ab-ELISA BRSV Kit, BIO K 061/2, Rochefort, Belgium). This test uses recombinant F protein (F) of BRSV for the detection of monoclonal antibodies specific to the F protein of BRSV. The test protocol was applied as recommended by the manufacturers. The optical density was measured using a spectrophotometer (Spectra SLT Classic, Virginia, Wietzendorf, Germany) at 450 nm, and the results were evaluated as described by the manufacturer.

### 2.9. Virus Isolation

Three nasal/oropharyngeal swabs and one lung sample that were found to be positive for BRSV and had low Ct values (below 26) per real-time RT-PCR were used for virus isolation. The swab samples were first diluted 1:2 using DMEM (Gibco, Cat. No: 21969035, Paisley, UK) containing 1% penicillin/streptomycin, then homogenized by vortexing. Lung tissue samples (0.5 g) in transport medium were cut into small pieces, placed in a sterile mortar, and homogenized with 5 mL of DMEM containing 1% penicillin/streptomycin. The homogenate was then centrifuged and the supernatant was filtered through a 0.22-µm filter (Aisimo, Cat. No: ASF33PVI22S, Shanghai, China). This filtrate (0.5 mL) was inoculated into each well of Madin-Darby bovine kidney (MDBK) cells cultured in six-well plates and incubated for 1 h at 37 °C, then the inoculations were discarded and 3 mL DMEM containing 1% penicillin/streptomycin was added. MDBK cells (ATCC CLL 22) were checked daily for the presence of cytopathic effects (CPE), particularly for syncytium formation. After 7 days of inoculation, the cell cultures were frozen and thawed and supernatants were transferred to a newly cultured MDBK cell monolayer. Blind passages were performed up to the third cell passage. In each passage, the supernatant was analysed for the presence of BRSV-RNA using real-time RT-PCR as described above.

### 2.10. Statistical Analyses

The distribution of ELISA OD values was applied to histogram analysis in the Minitab software program (Minitab, Version 19.2020.2.0, Chicago, IL, USA). It was observed that there were groupings in two fields in the histogram data; for this, the Grubbs test and boxplot were drawn in the MedCalc program and “outlier values control” was performed.

Statistical analysis of the relationship between positivity and the vaccinated and non-vaccinated status of animals found to be positive by ELISA and real time RT-PCR was performed using the Chi-Square test in the MedCalc software program (MedCalc, Version 20.218, Ostend, Belgium). *p* values of less than <0.05 were considered statistically significant.

## 3. Results

### 3.1. Clinical and Necropsy Findings

**Figure 1:**
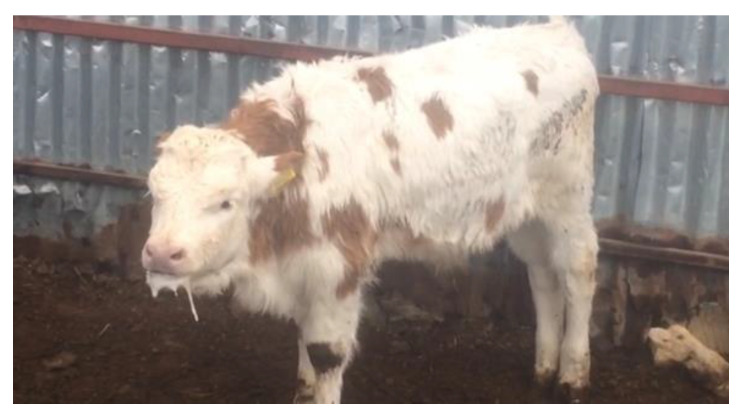
Clinical picture of calf with severe pneumonia, positive for BRSV-RNA by real-time RT-PCR, showing dyspnea, increased respiratory rate, cough, and frothy discharge from the mouth.

Clinical findings, gross pathology of the lungs, and information about the farms and animals analysed in this study are provided in Table 1. In animals from which nasal/oropharyngeal swabs (277) and blood (162) samples were taken, one or more of symptoms such as fever, fatigue, depression, respiratory distress, cough, and serous eye and nasal discharge were observed as clinical findings (Table 1). Wheezing, dyspnea, labored breathing, increased respiratory rate, and foamy discharge from the mouth were observed in a calf that had severe pneumonia and was positive for BRSV by real-time RT-PCR (Figure 1).

**Figure 2:**
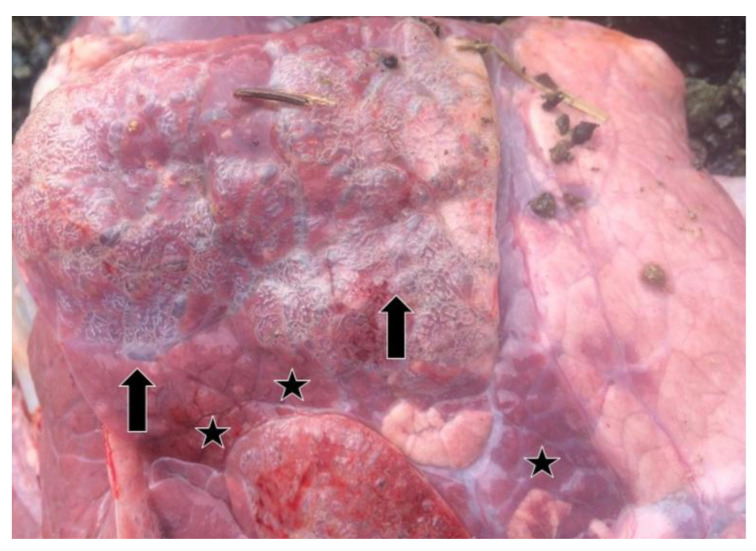
Air blebs (arrows), areas of consolidation (stars), and diffuse interstitial emphysema in the interlobular and subpleural regions in the lung of a BRSV-RNA positive calf as seen at necropsy.

Relevant information about 37 lung samples taken from animals which died as a result of severe pneumonia and from cattle with pneumonia before or after slaughter in the abattoir are provided in Table 1. In the lung samples which were positive by real-time RT-PCR, consolidation, areas of local or widespread congestion, edema, and interlobular or interstitial emphysema were present at necropsy (Figure 2).

### 3.2. Seropositivity of BRSV Detected by ELISA

**Figure 3:**
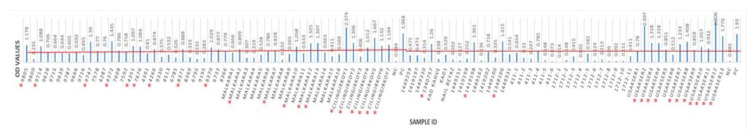
ELISA cutoff and OD values of non-vaccinated cattle. The red line indicates the border line for the cutoff value. Seropositive animals are marked with red star *.

**Figure 4:**
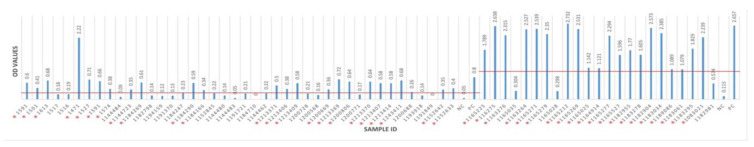
ELISA cutoff and OD values of vaccinated cattle. The red line indicates the border line for the cutoff value. Seropositive animals are marked with red star *.

**Figure 5:**
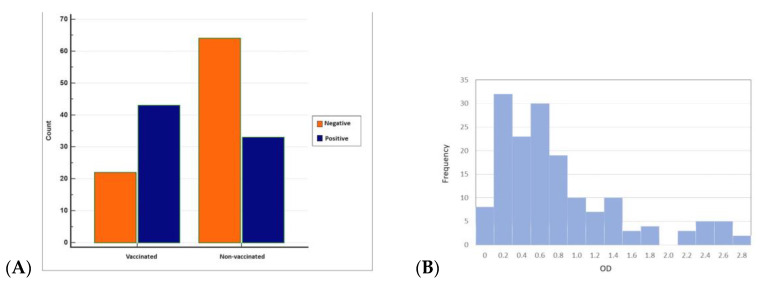
(**A**) Distribution of negative and positive ELISA OD values of vaccinated and non-vaccinated cattle (MedCalc) and (**B**) histogram showing the number of animals in the corresponding ELISA OD values of vaccinated and non-vaccinated cattle (Minitab).

Overall, antibodies to BRSV were detected in 76 (46.91%) of 162 sera from 62 herds having BRSV-vaccinated and non-vaccinated calves and young cattle with respiratory system disorders (Figure 3 and Figure 4). BRSV seropositivity was detected in 33 (34%) of 97 sera from non-vaccinated animals (Figure 3) and 43 (66%) of 65 sera from vaccinated animals (Figure 4). The distribution of negative and positive ELISA OD values of vaccinated and non-vaccinated cattle are provided in Figure 5A,B. Of the 33 positive sera from non-vaccinated animals, 11 included samples from Usak and 22 from the Thrace region, which borders the European Union. Eight sera belonged to calves in the 6–12 months age group and 25 sera were from the 0–6 months age group. All of the 43 positive sera from vaccinated animals were from the Thrace region, 13 belonged to calves in the 6–12 months age group and 30 sera were from the 0–6 months age group.

When ELISA OD data were analysed with histogram analyses by the Chi-squared test to compare seropositives and seronegatives, the difference between the vaccinated and non-vaccinated groups was found to be statistically significant (*p* < 0.05).

### 3.3. Efficiency of Real Time RT-PCR and Detection of BRSV-RNA in Clinical Samples

**Figure 6:**
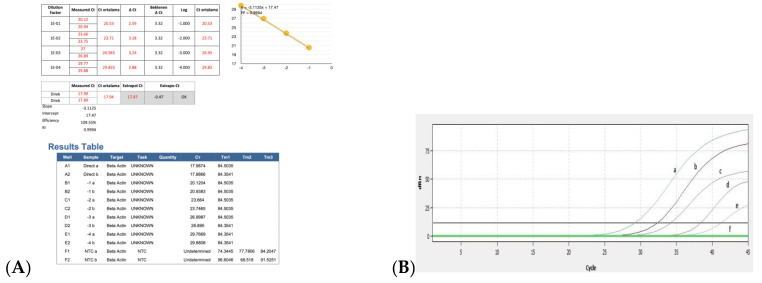
Ct values obtained using serial dilutions of positive control for PCR efficiency: (**A**) results of PCR efficiency and (**B**) amplification curves obtained after serial dilutions of positive control by real time RT-PCR. a, positive control (neat); b, 10^−1^ dilution of positive control; c, 10^−2^ dilution of positive control; d, 10^−3^ dilution of positive control; e, 10^−4^ dilution of positive control; f, negative control.

The results for the PCR efficiency using the β-actin gene and real-time RT-PCR threshold cycle (Ct) values obtained by serial dilutions of positive control are shown in Figure 6A,B. When ten-fold dilution of the positive control sample was used to determine the real time RT-PCR efficiency, the Ct values were obtained as 29, 32, 35, 38, and 41, respectively. Ct was detected up to 10^−4^ dilutions with positive control, while no Ct was detected in the negative control (Figure 6B).

**Figure 7:**
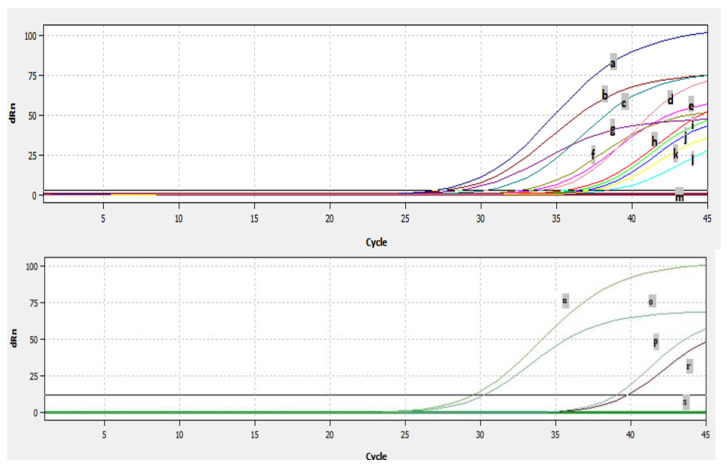
The Ct values of the positive samples: a, 9716 swab; b, 4351 swab; c, 7578 swab; d, 4343 lung; e, 4346 lung; f, 2601-12 swab; g, positive control; h, 4372 swab; i, sep lung; j, 1501 swab; k, 7592 swab; l, 4391 swab; m, negative control; n, usak lung; o, positive control; p, usak os1 swab; r, usak os3 swab; s, negative control.

Analyses of 277 nasal/oropharyngeal swabs and 37 lung samples by real-time RT-PCR showed that a total of fourteen samples, ten swabs, and four lung samples were found to be positive for BRSV by real-time RT-PCR (Figure 7). The fourteen samples which were detected as positive by real time RT-PCR originated from six different farms. The Ct of the positive samples ranged from 26–38 (Figure 7). BRSV-RNA was detected in eight of the nasal/oropharyngeal swabs collected from the farms in Thrace and in three of the lungs (Figure 7), and was detected in two of the nasal/oropharyngeal swabs taken from Usak and one of the lungs (Figure 7).

**Figure 8:**
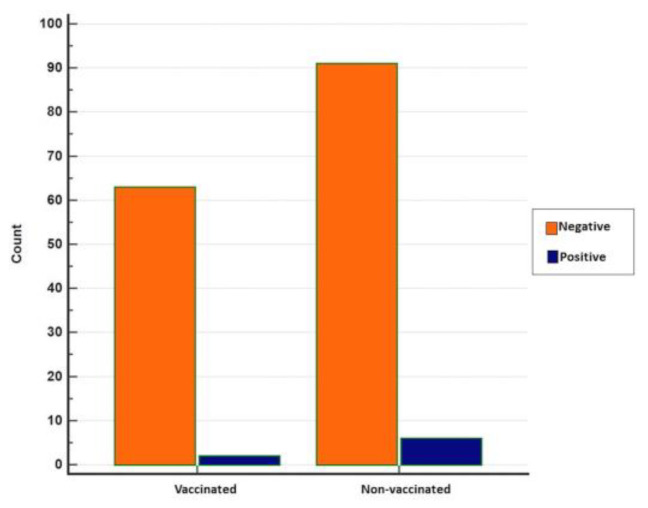
Distribution of negative and positive real-time RT-PCR results in vaccinated and non-vaccinated cattle (MedCalc).

The results of the statistical analyses show no statistical significance between vaccinated and non-vaccinated animals concerning real-time RT-PCR positivity (*p* = 0.3722), although positivity was relatively higher in non-vaccinated animals (Figure 8).

### 3.4. Sequencing and Phylogenetic Analysis

**Figure 9:**
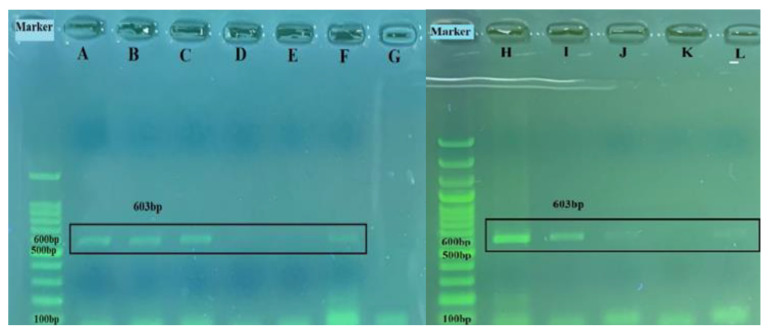
The 603 bp amplified products of the partial BRSV-G gene on horizontal gel electrophoresis: A, 7 578-swab; B, 9716-swab; C, 4343-lung; D, 4346-lung; E, 4351-swab; F, positive control; G, negative control; H, usak lung; I, usak os1 swab; J, usak os3 swab; K, negative control; L, positive control.

Among the ten nasal/oropharyngeal swabs and four lung samples found to be positive by real-time RT-PCR, a 603 base-pair band corresponding to the expected size of the BRSV-G gene was seen on agarose gel electrophoresis in four nasal/oropharyngeal swabs and one lung sample (Figure 9) after amplification by RT-PCR. Sequences of these samples were compared with other BRSV-G gene sequences containing 247 nucleotides submitted to GenBank using the Geneious Prime program in order to determine the percentage of similarity. The obtained data on the percentages of similarity are provided in Table 3.

**Figure 10:**
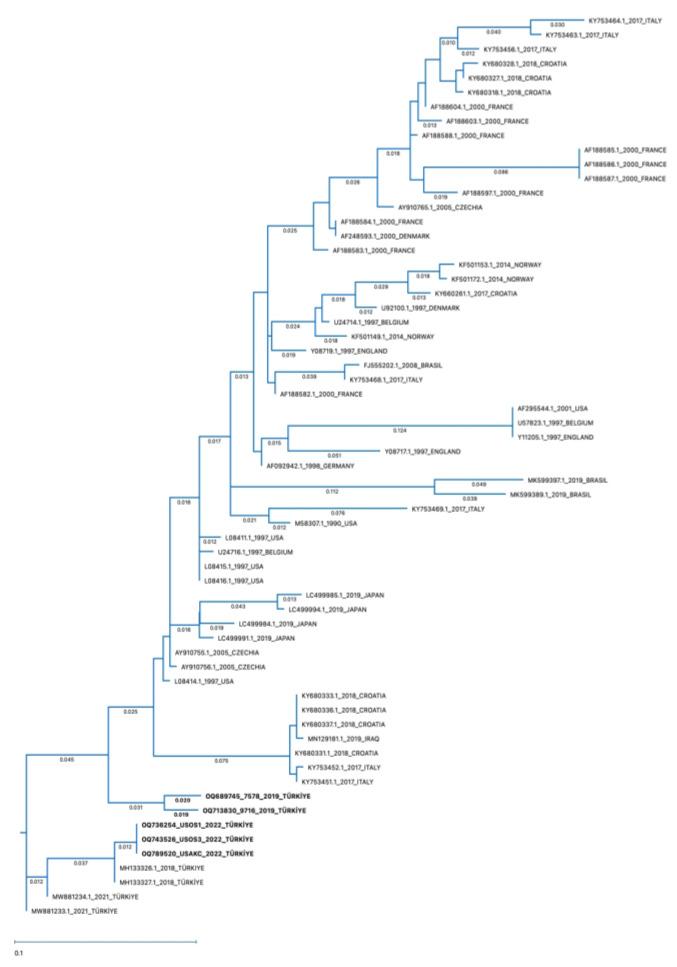
Phylogenetic tree of partial G genes of representative BRSVs. The Maximum Likelihood:RAxML method was used to construct the phylogenetic tree with 1000 Bootstrap replicates using MegAlign Pro Software (DNASTAR), using the G gene sequences of the strains detected in this study (OQ736254-swab, OQ743526-swab, OQ789520-lung, OQ689745-swab, and OQ713830-swab) and representative BRSV strains reported elsewhere. The accession numbers written in bold characters are the BRSVs detected in this study.

According to the phylogenetic tree, the identified BRSV-G gene sequences in the present study belong to Subgroup-III (Figure 10). The sequences of amplified positive samples in this study demonstrated 88–100% identity. The percentage similarity between two nasal/oropharyngeal swab and one lung sample obtained from the same farm and positive by RT-PCR was 88–100% (Table 3: OQ736254-swab, 743526-swab, and OQ789520-lung). The similarity rate of two nasal/oropharyngeal swab lung samples taken from the same farm was found to be 96% (Table 3: OQ689745-swab and OQ713830-swab). The sequence in the BRSV-positive lung sample was found to be 97.17% similar to the BRSV genotype in the lung sample previously detected in our country and reported to GenBank (Table 3: MH133326.1). It was found that four positive nasal/oropharyngeal swab samples were 89–98% similar to the BRSV-G gene sequences previously detected in our country and reported to GenBank (Table 3: MH133326.1, MH133327.1, MW881233.1, MW881234.1).

When compared with the BRSV genotypes detected in other countries, the highest similarity of 89.47–93.12% was found between the genotype reported in the USA (Table 3: L08414.1) and the genotype reported in Czechia (Table 3: AY910755.1). There was 86.23–89.07% similarity between the gene sequences of five positive samples and the genotype reported to GenBank from Iraq, our neighboring country (Table 3: MN129181.1). The lowest similarity rate (78.95%) was found among the genotype detected in Brazil (Table 3: MK599389.1).

The gene region containing 247 nucleotides from the BRSV-G gene partial sequence analysis of four swabs and one lung which were found to be positive as a result of RT-PCR analysis was at least three nucleotides compared to the BRSV-G gene sequences previously reported to GenBank from our country, and sequence differences containing up to 26 nucleotides were found, while 44–52 nucleotide differences were detected with the genotype detected in Brazil (Table 3: MK599389.1), which had the lowest similarity rate. When the genotype sequence detected in our neighboring country of Iraq (Table 3: MN129181.1) was compared, 30–34 nucleotide differences were found.

### 3.5. Virus Isolation

**Figure 11:**
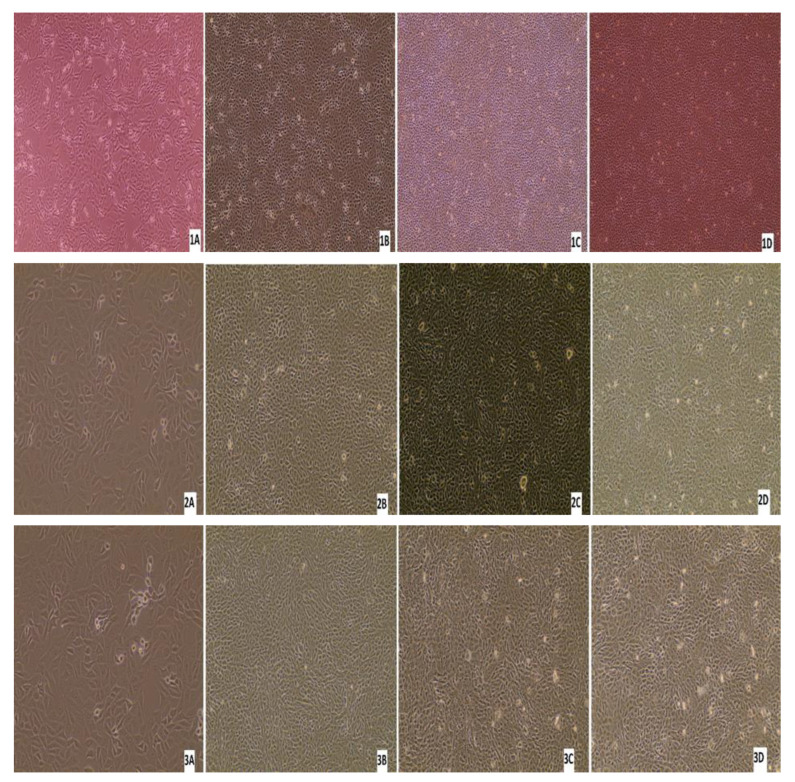

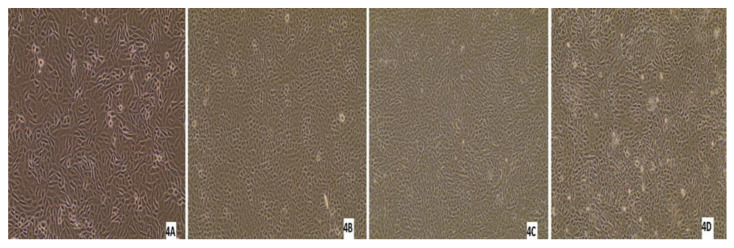
Appearance of MDBK cells after inoculations of the samples: (**1A**) confluent MDBK cells; (**1B**) swab sample-7578 at 7 days after inoculation of (first blind passage); (**1C**) second blind passage (day 7); (**1D**) third blind passage (day 7); (**2A**) appearance of MDBK cells after inoculation of the samples; (**2B**) swab sample-9716 at 7 days after inoculation (first blind passage); (**2C**) second blind passage (day 7); (**2D**) third blind passage (day 7); (**3A**) confluent MDBK cells; (**3B**) swab sample-7578 at 7 days after inoculation (first blind passage); (**3C**) second blind passage (day 7); (**3D**) third blind passage (day 7); (**4A**) confluent MDBK cells; (**4B**) swab sample-7578 at 7 days after inoculation (first blind passage); (**4C**) second blind passage (day 7); (**4D**) third blind passage (day 7) (Evos XL Core, 10×).

Three nasal swabs and a lung sample with a low Ct value (below 26) per real-time RT-PCR were subjected to virus isolation. From the three animals with virus-positive swabs, only one animal was antibody-positive by ELISA, while the other two were antibody-negative. Monolayer MDBK cells were inoculated with 200 μL of the sample/well. After 7 days of inoculation, if there was no CPE, three blind passages were carried out. No CPE was detected in any of the samples after the first inoculation or after each passage. After each blind passage, the cell culture supernatant was collected and analysed by real-time RT-PCR for BRSV. BRSV-RNA was detected in only one swab sample after 7 days from the first inoculation, and no CPE was detected in these cells (Figure 11). All the other cell supernatants taken during cell culture studies were negative by real-time RT-PCR, there was no CPE in the control cells, and no Ct was detected in culture supernatant by real-time RT-PCR.

## 4. Discussion

Bovine respiratory syncytial virus infection causes serious respiratory tract infections in calves and young cattle and economic losses in many countries, including Turkey [36,41]. BRSV infection especially affect young animals, spreads rapidly among non-vaccinated animals, and can cause serious respiratory disorders such as pneumonia, especially in the presence of other respiratory pathogens causing bovine respiratory disease complex (BRDC) [2,12]. Therefore, this study investigated the frequency, antibody response, molecular diversity, and circulating genotypes of BRSV in cattle on farms located in Turkey’s Usak province and Thrace district, which borders the European Union.

BRSV has been causing outbreaks in Europe and other continents since 1970 [20]. In the present study, antibody response to vaccination and infection was investigated using ELISA. Other studies have detected the seroprevalance of BRSV in Europe as between 11.6% to 100%; the rate was 11.6% in Spain [42], 69.1% in Italy [10], and 100% in Ireland [43,44]. It has been emphasized that low prevalence is correlated with the presence of maternal antibodies, while high prevalence (90%) is associated with a history of pneumonia [44]. These reports show that cases with pneumonia should be examined for BRSV. Similarly, most of the non-vaccinated animals in which antibodies were detected in the present study were those with clinical pneumonia.

In the USA, BRSV antibodies have been detected at a level of 69.5% in the southern US, while the same rate was found to be 40.9% in the northern US [45]. Similar to the USA, studies on seroprevalence carried out in South American countries found seropositivity to be 78.64% in Argentina [46] and 79.5% in Brazil [33].

A number of studies have been performed on BRSV seroprevalence in Turkey. The first study reported 46.12% seropositivity in non-vaccinated cattle [47]. Non-vaccinated cattle sera were analysed in another study in Turkey, which found seropositivity of 44.6% in Central Anatolia [48], 58.48% in Afyonkarahisar and Usak provinces [27], 67.3% in Eastern and Southeastern Anatolia [49], 73% in Marmara Region [50], 76.38% in Denizli and Burdur provinces [51], 97.1%in Adiyaman province [52], and 98.6% in Aydın province [51]. In neighboring countries, BRSV seroprevalence in non-vaccinated animals was found to be 83.11% in Iraq [53] and 51.1% [54] and 89.1% in two studies from Iran [55].

According to the data of the studies reported above, both low and high seropositivity have been detected in Turkey as well as in other countries. In the present study, the overall seroprevalence in 162 vaccinated and non-vaccinated sera was found to be 46.9%. This value is higher than the seroprevalence values found in Spain and the northern US, and is lower than the southern US, Italy, Ireland, Serbia, Argentina, Brazil, Iraq, and Iran. Seroprevalence studies in our country were performed with serum samples from animals that were not vaccinated against BRSV. The seroprevalence (34%-33 of 97 sera) found in the non-vaccinated cattle in the present study was lower than the seropositivity values previously determined in our country [47,48,49,50,51,52]. In the present study, BRSV antibodies were detected in 43 samples (66%) out of 65 sera from vaccinated cattle. It is thought that the majority of this positivity detected in vaccinated animals is due to the antibodies formed as a result of the vaccination. In order to determine whether the BRSV antibodies detected in vaccinated animals are due to the vaccine or to an infection, paired serum samples taken at 3–4 weeks intervals should be used and the seroconversion should be checked. However, this was not the target of our study.

It is not surprising that vaccinated animals had higher levels of BRSV-specific serum antibodies than nonvaccinated animals. Because there is no DIVA (differentiating infected from vaccinated animals) BRSV vaccine available as of yet, we cannot differentiate vaccinated from infected animals. The only conclusion that we can draw from this is that the “vaccinated” group was most likely vaccinated with a BRSV vaccine. The seronegative animals within the “vaccinated” group were most likely vaccinated some time ago and in need of a booster vaccination.

In addition to serology, we analysed nasal/oropharyngeal swabs and tissue samples for the presence of BRSV-RNA using real-time RT-PCR and RT-PCR, as previously used by others [31,41,56,57,58]. In this study, rapid and specific detection of BRSV-RNA, probability-based real-time RT-PCR targeting the N gene of BRSV was used [39,56,58,59]; however, other genes, such as the F and G genes, have been targeted to detect BRSV-RNA in previous studies [60,61]. In the present study, 277 nasal/oropharyngeal swabs and 37 lung samples were analysed by real-time RT-PCR, with ten swabs (3.6%) and four lungs (10.8%) found to be positive for BRSV-RNA. The difference in detection between swabs and lung samples could be due to the different levels of viral load in swabs and lungs or to the disease stage of the animals, i.e., whether they were acutely or chronically infected.

Both low and high detection rates of BRSV infection in cattle have been reported in other countries using real-time RT-PCR [57,62]. Concerning Europe, in Italy all nasal/oropharyngeal swabs, bronchoalveolar lavage samples, and lung samples were positive for BRSV-RNA [34]. In another study, 14.5% of 138 oropharyngeal swabs were positive in cattle transferred from France to Southern Italy [63]. BRSV-RNA was detected in 29.4% of 128 bronchoalveolar fluid samples in Belgium [64], 21% of 764 nasal swabs in Sweden [62], 86% of 21 respiratory tract samples in Norway [57], and 5.1% of 541 nasal swabs and bronchoalveolar lavage samples in the United Kingdom [31]. In the USA, 3.79% of 3215 nasal and oropharyngeal swabs samples [32] and 9.1% of 122 animals [65] were found to be positive for BRSV-RNA. The data obtained in the US are similar to those found in the present study. However, when the data from European countries are compared with the present study, it is noteworthy that lower positivity was detected in this study. This may be due to the fact that most of the animals sampled in this study were vaccinated, and vaccination may lead to low level of shedding and circulation of BRSV among cattle. This point is important in terms of prevention and control of BRSV in cattle.

Findings with PCR in other studies performed in Turkey have been both similar and different compared to those obtained in the present study [27,36,37]. In one previous study in Erzurum, Turkey, 1.29% of 155 nasal swabs were positive for BRSV-RNA [36]. The rate of positivity found in this study is higher than the detection rate found by Timurkan and others [36]. However, in a study performed in 2018 in Samsun, Turkey, three lugs were analysed and all samples were positive for BRSV-RNA [37]. In addition, 2 of 21 nasal swabs were positive for BRSV-RNA in Afyonkarahisar and Usak, representing a low level of BRSV circulation [27]. Higher detection rates were reported in the neighboring countries Iraq (37.31%) [53] and Iran (78.12%) [66].

The varying level of detection in our country and other countries could be due to the age of animals, their vaccination and clinical status, the time of sampling (presence of clinical signs), application of the diagnostic tests, and differences in virus circulation levels in different farms and geographic areas. Although all these factors affect the frequency of BRSV in cattle, vaccination is an important practice to reduce the virus frequency and circulation of BRSV in the field, as was seen in this study. In the present study, when nasal/oropharyngeal swab samples of 43 vaccinated animals found to be seropositive by ELISA were examined by real-time RT-PCR, BRSV-RNA was detected in two samples. On the other hand, when nasal/oropharyngeal swab samples of 33 non-vaccinated animals which were found to be seropositive by ELISA were examined by real-time RT-PCR, BRSV-RNA was detected in six samples. Although the correlation of BRSV-RNA detection in vaccinated and non-vaccinated animals was not statistically significant, the proportion of BRSV-RNA positivity in non-vaccinated cattle was higher than in vaccinated cattle.

Sequence analysis studies on BRSV have either targeted the whole genome or partial/whole genes of interest. Next-generation sequencing techniques have been used for whole genome analysis in recent years. The F [12,36], N [6,34], and G gene [8,41,67] of BRSV are frequently targeted to investigate mutations in these genes for sequence analysis; because mutations in the G gene are more frequent than in the F gene, there are more studies on sequencing the BRSV-G gene. Therefore, the BRSV-G gene was targeted in this study to investigate circulating genotypes and genotypic diversity. Phylogenetic analyses using the BRSV-F and BRSV-G gene sequences resulted in a total of ten genotypically different subgroups of BRSV [7,8,20].

Subgroup-II genotypes have been reported to circulate in Norway [57] and Japan [8]. According to the data of recent studies, genotypes belonging to Subgroup-III have been found to circulate in Turkey [27] and in the USA [26]. BRSV genotypes detected in western Europe are generally included in Subgroup-V and Subgroup-VI [25]. In the present study, partial G gene sequences of BRSV from four nasal/oropharyngeal swab samples and one lung sample indicated that all genotypes belonged to Subgroup-III. It was found that while all of the partial G gene sequences were similar, there was some degree of diversity compared to those previously reported in Turkey. In a previous study performed in Eskisehir, Turkey, two lung samples were reported to be in BRSV Subgroup-III [67]. These were 97.17% similar to the lung sample found in this study. Similarly, BRSV Subgroup-III was found in two nasal swab samples from Erzurum, Turkey [36]. Two nasal swab samples detected in Usak, Turkey were characterised as BRSV Subgroup-III [27], and were 89–95% similar to the nasal/oropharyngeal samples found in this study. Five BRSV genotypes detected in this study were similar to the BRSV genotypes detected in other countries, and especially with genotypes reported in the USA [68] and Czechia [25]. It was observed that the highest similarity with the genotypes was 89.47–93.12% [25]. The lowest similarity rate was found among the genotype detected in Brazil [69].

The results of other studies have revealed that isolation of BRSVs is rather difficult for a number reasons, including the requirement for many passages, cell types, cell specificity–affinity, the difficulty of attaching and adaptation to cells [70], the clinical stage of BRSV-infected animals, the type of samples, and the viral load of samples [1,71,72]. In the present study, BRSV isolation was attempted using samples from the clinically ill animals; however, BRSV could not be isolated from any samples using MDBK cells even though three blind passages were made. Similar results have been obtained in other studies, where no CPE was seen in cell cultures used for virus isolation [73,74]. Yazıci and others [37] reported CPE after 72 h of incubation in MDBK cell cultures, whereas Arns and others [72] were able to detect CPE in MDBK cells in only 1 of 33 samples after nine blind passages. In the present study, we could not see CPE after three passages and failed to detect BRSV-RNA in the culture supernatants.

The limitation of virus isolation is the low number of repassages, as three passages seem to not be enough. However, as discussed above, both three repassages and higher numbers of repassages have been used in other studies [72,73,74]. Another limitation of our study is that we could not differentiate vaccinated from infected animals, as DIVA BRSV vaccines are not available yet. Paired serum samples taken at 3–4 week intervals could have been taken to check seroconversion in vaccinated and non-vaccinated animals, which could help to differentiate infected from vaccinated; however, this was not aim of our study. Another limitation was that we only amplified a portion of the BRSV genome (the G protein gene) and used this for our phylogenetic analyses; however, we believe that these limitations did not have a significant impact on our results or their interpretations.

In conclusion, BRSV infection continues to be a threat to cattle production in Turkey, despite vaccinations. It is beneficial to continue monitoring, vaccinations, and eradication studies for BRSV infections. The results of this study show that antibody positivity in vaccinated animals was statistically significant and that the detection rate of BRSV-RNA in these animals was lower than in non-vaccinated animals. The integrated farms and small public farms should have a vaccination strategy, and interference of vaccine strains with maternally-derived antibodies should be taken into account. In addition, the diversity of the BRSVs found in this study should be considered in vaccination strategies.

## Figures and Tables

**Table 1:** Date of sample collection, sample type, locality, age and number of animals, and clinical signs/pathology of the animals analysed in this study.

Sample Collection Date	Sample Region	Age (Months)	Number of Samples			Clinical Signs/Pathology
			Blood	Swabs	Lungs	
21 January 2019	Luleburgaz	0–6	10	10		Dyspnea, cough, serous nasal secretions, fatigue
20 February 2019	Luleburgaz	0–6	2	2		Dyspnea, cough, serous nasal secretions, fatigue
20 February 2019	Catalca-Istanbul	0–6		18		Dyspnea, cough, serous nasal secretions, fatigue
21 February 2019	Luleburgaz	0–6	11	11	2	Dead calf
26 February 2019	Usak	0–6		20		Dyspnea, cough, serous nasal secretions, fatigue
13 January 2019	Malkara	0–6	18	18		Dyspnea, cough, serous nasal secretions, fatigue
14 January 2019	Kirklareli	0–3	9	9		Cough, serous nasal secretions, fatigue
26 January 2019	Kirklareli	0–6		25		Dyspnea, cough, serous nasal secretions, fatigue
10 February 2019	Edirne	0–3	15	15		Cough, serous nasal secretions, fatigue
13 February 2019	Cilingirkoy-Istanbul	0–12	11	10		Fever and cough
22 February 2019	Luleburgaz	0–6		19		Dyspnea, cough, serous nasal secretions, fatigue
23 February 2019	Kirklareli	0–12	3	1		Dyspnea, cough, serous nasal secretions, fatigue
24 February 2019	Kirklareli	0–6		5		Dyspnea, cough, serous nasal secretions, fatigue
4 March 2021	Luleburgaz	0–6	25	25	1	Dead calf
9 March 2021	Kirklareli	0–6		4		Dyspnea, cough, serous nasal secretions, fatigue
18 March 2021	Kirklareli	0–6	9	9		Dyspnea, cough, serous nasal secretions, fatigue
2 April 2021	Kirklareli	0–6		5		Dyspnea, cough, serous nasal secretions, fatigue
2 April 2021	Buyukcekmece-Istanbul	6–18			5	Abattoir sample: Consolidation, hyperemia
7 April 2021	Cilingirkoy-Istanbul	0–12	7	7		Fever and cough
19 April 2021	Edirne	0–12	12	12		Cough, serous nasal secretions, fatigue
7 April 2021	Balikesir	12–24			19	Abattoir sample: Consolidation, hyperemia
4 November 2021	Edirne	0–6	6	6		Cough, serous nasal secretions, fatigue
17 December 2021	Edirne	0–6	11	11		Cough, serous nasal secretions, fatigue
5 January 2022	Esenyurt-Istanbul	12–24			9	Abattoir sample: Consolidation, hyperemia
20 January 2022	Usak	0–6	3	3	1	Dead calf Cough, serous nasal secretions, fatigue
1 March 2022	Edirne	0–3		2		Cough, serous nasal secretions, fatigue
4 March 2022	Usak	0–6		20		Cough, serous nasal secretions, fatigue
7 March 2022	Kirklareli	0–3	10	10		Fever, serous nasal secretions, fatigue
Total			162	277	37	

**Table 2:** Primers and probe used for detection and sequencing of BRSV.

Primers and Probe	Target Genes	Primers and Probe Sequences	Size (bp)	Positions	References
B5A-B6A RT-PCR	G gene	F: 5′-CCACCCTAGCAATGATAACCTTGAC-3′ R: 5′-AAGAGAGGATGC(T/C)TTGCTGTGG-3′	603	110–134 691–712	[29]
BRSV Real time RT-PCR	N gene	F: 5′-GCAATGCTGCAGGACTAGGTATAAT-3′ R: 5′-ACACTGTAATTGATGACCCCATTCT-3′	123	977–1001 1076–1100	[39]
Probe		FAM-5-ACCAAGACTTGTATGATGCTGCCAAAGCA-3-TAMRA		1028–1056	[39]

**Table 3:** Percentage homology of the nucleotide sequences among bovine respiratory syncytial virus strains detected in different countries and in Turkey based on partial (603 bp) G gene analysis (MegAlign Pro Software (DNASTAR).

Distance/ Distance	OQ713830_9716 2019_TÜRKİYE	OQ689745_7578 2019_TÜRKİYE	OQ736254_USOS1 2022_TÜRKİYE	OQ743526_USOS3 2022_TÜRKİYE	OQ789520_USAKC 2022_TÜRKİYE	Distance/ Distance	OQ713830_9716 2019_TÜRKİYE	OQ689745_7578 2019_TÜRKİYE	OQ736254_USOS1 2022_TÜRKİYE	OQ743526_USOS3 2022_TÜRKİYE	OQ789520_USAKC 2022_TÜRKİYE
OQ713830_9716_2019_TÜRKİYE	0.00	96.36	89.88	89.88	88.26	KY753468.1_2017_ITALY	87.85	87.04	87.04	87.04	85.43
OQ689745_7578_2019_TÜRKİYE	96.36	0.00	89.07	89.07	87.45	M58307.1_1990_USA	87.45	87.45	86.64	86.64	85.02
AY910755.1_2005_CZECHIA	92.71	93.12	91.50	91.50	89.88	Y08719.1_1997_ENGLAND	87.45	88.26	87.45	87.45	85.83
L08414.1_1997_USA	92.71	93.12	90.69	90.69	89.47	AY910765.1_2005_CZECHIA	87.04	85.83	87.04	87.04	85.43
AY910756.1_2005_CZECHIA	92.31	92.71	91.09	91.09	89.47	KF501149.1_2014_NORWAY	87.04	87.85	86.23	86.23	84.62
L08416.1_1997_USA	91.90	92.31	90.69	90.69	89.07	FJ555202.1_2008_BRASIL	87.04	86.23	86.23	86.23	84.62
L08415.1_1997_USA	91.90	92.31	90.69	90.69	89.07	AF188583.1_2000_FRANCE	87.04	87.04	87.04	87.04	85.43
MW881233.1_2021_TÜRKİYE	91.09	91.90	93.93	93.93	92.31	Y08717.1_1997_ENGLAND	86.64	86.23	86.64	86.64	85.02
U24716.1_1997_BELGIUM	91.09	91.50	90.69	90.69	89.07	U92100.1_1997_DENMARK	86.23	86.23	86.23	86.23	84.62
LC499991.1_2019_JAPAN	91.09	92.31	89.88	89.88	88.26	AF188588.1_2000_FRANCE	85.83	85.43	85.83	85.83	84.21
L08411.1_1997_USA	90.69	91.09	89.47	89.47	87.85	KY660261.1_2017_CROATIA	85.43	85.43	83.81	83.81	82.19
MH133326.1_2018_TÜRKIYE	90.28	89.47	98.79	98.79	97.17	KY753469.1_2017_ITALY	85.43	83.81	84.62	84.62	83.00
MH133327.1_2018_TÜRKİYE	90.28	89.47	98.79	98.79	97.17	AF188604.1_2000_FRANCE	85.43	85.02	86.23	86.23	84.62
OQ736254_USOS1_2022_TÜRKİYE	89.88	89.07	0.00	100.00	98.38	AF188603.1_2000_FRANCE	84.62	84.21	85.43	85.43	83.81
OQ743526_USOS3_2022_TÜRKİYE	89.88	89.07	100.00	0.00	98.38	KY753463.1_2017_ITALY	84.62	85.02	83.81	83.81	82.19
MW881234.1_2021_TÜRKiYE	89.88	90.69	95.14	95.14	93.52	KF501172.1_2014_NORWAY	84.21	85.02	82.59	82.59	80.97
KY680331.1_2018_CROATIA	89.88	88.66	88.66	88.66	87.04	KY753456.1_2017_ITALY	84.21	83.81	85.02	85.02	83.40
LC499984.1_2019_JAPAN	89.88	90.28	89.47	89.47	87.85	KY753464.1_2017_ITALY	84.21	83.81	83.00	83.00	81.38
KY680337.1_2018_CROATIA	89.47	88.26	88.26	88.26	86.64	KY680318.1_2018_CROATIA	84.21	84.62	84.21	84.21	82.59
KY680336.1_2018_CROATIA	89.47	88.26	88.26	88.26	86.64	KF501153.1_2014_NORWAY	83.81	85.43	82.19	82.19	80.57
KY680333.1_2018_CROATIA	89.47	88.26	88.26	88.26	86.64	AF188597.1_2000_FRANCE	83.81	83.40	83.81	83.81	82.19
KY753451.1_2017_ITALY	89.47	88.26	88.26	88.26	86.64	MK599397.1_2019_BRASIL	83.81	84.21	82.59	82.59	80.97
MN129181.1_2019_IRAQ	89.07	87.85	87.85	87.85	86.23	KY680327.1_2018_CROATIA	83.40	83.81	84.21	84.21	82.59
AF092942.1_1998_GERMANY	89.07	89.07	87.45	87.45	85.83	KY680328.1_2018_CROATIA	83.00	83.40	83.81	83.81	82.19
KY753452.1_2017_ITALY	89.07	87.85	88.66	88.66	87.04	Y11205.1_1997_ENGLAND	82.59	81.38	81.38	81.38	79.76
LC499985.1_2019_JAPAN	89.07	88.66	87.85	87.85	86.23	U57823.1_1997_BELGIUM	82.59	81.38	81.38	81.38	79.76
OQ789520_USAKC_2022_TÜRKİYE	88.26	87.45	98.38	98.38	0.00	AF295544.1_2001_USA	82.59	81.38	81.38	81.38	79.76
AF248593.1_2000_DENMARK	88.26	88.26	87.45	87.45	85.83	MK599389.1_2019_BRASIL	82.19	83.40	80.57	80.57	78.95
AF188584.1_2000_FRANCE	88.26	88.26	87.45	87.45	85.83	AF188585.1_2000_FRANCE	81.38	81.78	82.19	82.19	80.57
AF188582.1_2000_FRANCE	88.26	88.26	86.64	86.64	85.02	AF188586.1_2000_FRANCE	81.38	81.78	82.19	82.19	80.57
LC499994.1_2019_JAPAN	88.26	88.66	88.66	88.66	87.04	AF188587.1_2000_FRANCE	81.38	81.78	82.19	82.19	80.57
U24714.1_1997_BELGIUM	87.85	87.85	87.04	87.04	85.43						

## Data Availability

All data are included in the manuscript.
